# Subjective reasons why immigrant patients attend the emergency department

**DOI:** 10.1186/s12873-015-0031-8

**Published:** 2015-03-28

**Authors:** Ibrahim Mahmoud, Rob Eley, Xiang-Yu Hou

**Affiliations:** Department of Emergency Medicine, Royal Brisbane & Women’s Hospital – School of Medicine, The University of Queensland, and School of Public Health and Social Work, Queensland University of Technology, Brisbane, Australia; School of Medicine and the Department of Emergency Medicine– Princess Alexandra Hospital, The University of Queensland, Brisbane, Australia; School of Public Health and Social Work, Queensland University of Technology, Brisbane, Australia

**Keywords:** Emergency department, General practitioner, Immigrants

## Abstract

**Background:**

Some patients visit a hospital’s emergency department (ED) for reasons other than an urgent medical condition. There is evidence that this practice may differ among patients from different backgrounds. The objective of this study was to examine the reasons why patients from a non-English speaking background (NESB) and patients with an English speaking background but not born in Australia (ESB-NBA) visit the ED, as compared to patients from English-speaking backgrounds but born in Australia (ESB-BA).

**Methods:**

A cross-sectional survey was conducted at the ED of a tertiary hospital in metropolitan Brisbane, Queensland, Australia. Over a four-month period patients who were assigned an Australasian Triage Scale score of 3, 4 or 5 were surveyed. Pearson chi-square test and multivariate logistic regression analyses were performed to examine the differences between the ESB and NESB patients’ reported reasons for attending the ED.

**Results:**

A total of 828 patients participated in this study. Compared to ESB-BA patients NESB patients were less likely to consider contacting a general practitioner (GP) before attending the ED (Odds Ratios (OR) 0.6 (95% Confidence Interval (CI) 0.4–0.8, p < .05) While ESB-NBA were more likely to consider contacting a GP 1.7 (1.1–2.5, p < .05). Both the NESB patients and the ESB-NBA patients were far more likely than ESB-BA patients to report that they had visited the ED either because they do not have a GP (OR 7.9, 95% CI 4.7–13.4, p < .001) and 2.2 (95% CI 1.1–4.4, p < .05) respectively and less likely to think that the ED could deal with their problem better than a GP (OR 0.5 (95% CI 0.3–0.8, p < .05) and 0.7 (0.3–0.9, p < .05) respectively. The NESB patients also thought it would take too long to make an appointment to consult a GP (OR 6.2, 95% CI 3.7–10.4, p < 0.001).

**Conclusions:**

NESB patients were the least likely to consider contacting a GP before attending hospital EDs. Educational interventions may help direct NESB people to the appropriate health services and therefore reduce the burden on tertiary hospitals ED.

**Electronic supplementary material:**

The online version of this article (doi:10.1186/s12873-015-0031-8) contains supplementary material, which is available to authorized users.

## Background

Several international studies have suggested that immigrants, particularly those from non-English speaking backgrounds (NESB) might use emergency departments EDs inappropriately [1-3]. These studies identified several potential barriers confronting immigrants when accessing health care. These obstacles were suggested to include the ease of access to emergency services, language and cultural barriers, unawareness of service availability, and a lack of knowledge about other health care services in the new country. It has been argued that emergency department (ED) presentations for non-urgent conditions are less likely to involve preventive care and are costlier to the healthcare systems than visits to general practitioner (GP) clinics [4]. Moreover, such presentations may adversely affect people who are presenting at the same time with more urgent conditions through prolonged length of stay and increased wait times in EDs [2,5-7]. As a result the quality of the care provided in EDs might be reduced which can lead to an increased probability of complications [2,5-7].

The use of EDs for non-urgent conditions might also result in ED overcrowding which increases both patient dissatisfaction and the number of patients who leave the ED before being seen [8,9]. Consequently, it would be beneficial to reduce the demand for ED use and encourage patients to utilise primary care services for non-life threatening conditions.

In Australia, no studies have been conducted on either the reasons of non-English speaking background (NESB) patients for seeking medical care from the ED or the barriers they face when accessing health services in general. Understanding these reasons might help in developing policies and educational interventions that promote NESB patients’ access to appropriate health care facilities that meet their needs.

This study aimed to investigate the subjective reasons why immigrants to Australia (NESB patients and English speaking background not born in Australia (ESB-NBA)), compared to English-speaking background born in Australia (ESB-BA) patients, attended the tertiary hospital ED at Brisbane, Australia. An understanding of the reasons for seeking primary health care in the ED setting will support health service strategies.

## Methods

### Study design and setting

A cross-sectional survey was conducted in the ED of Princess Alexandra Hospital (PAH), an adult, tertiary-referral teaching hospital located in metropolitan south Brisbane, Queensland, Australia. The ED has an annual census in excess of adult 50,000 presentations. The majority of immigrants to Queensland live in metropolitan areas and the South Brisbane region is home to the largest number of people in Queensland who identify as being NESB [10,11].

The study was carried over a four-month period from 27 August to 28 December 2012. The data were collected by the main author, who attended the study hospital ED from Monday to Saturday between 12:00 pm and 8:00 pm. These times were chosen because, during this time period, patients are also more likely have a choice to go to a general practitioner (GP) or the ED.

Although private health is available in Australia medical treatment is free for all Australian citizens, New Zealand citizens, permanent residents in Australia, or those people who have applied for a permanent visa. All that is required is a Medicare card. By law public ED cannot charge for services. It is up to the discretion of GPs whether they charge in excess of what is covered by Medicare. For the vast majority of GPs an appointment must be made and this is usually one or two days in advance. In some locations GP books are full. In the ED onsite interpreter services and free telephone interpreter services are available. Only the latter may be available in GP clinics.

### Study population

A convenience sample was recruited. The inclusion criteria were patients aged 18 years and over, with Australasian Triage Scale (ATS) categories of 3 (urgent), 4 (semi-urgent) or 5 (non-urgent) [12]. The exclusion criteria were patients in triage categories 1 (critical) and 2 (emergency), those below 18 years of age, patients with dementia, mental health patients, pregnant women with obstetrical complaints and admitted patients.

The participants were divided into three groups as defined by the Australian Bureau of Statistics (ABS): patients born in Australia, patients born in English-speaking countries (predominately United Kingdom, New Zealand, Republic of Ireland, South Africa, the United Sates, and Canada,) and patients born in other countries where English is not the principal language [13]. These groups were named English speaking background born in Australia (ESB-BA), English speaking background not born in Australia (ESB-NBA) and Non English speaking background (NESB).

Arguing that being from an NESB did not indicate disadvantage, the federal government formally replaced ‘NESB’ with ‘culturally and linguistically diverse’ (CALD) in 1996 [14]. However, we chose to employ the old term ‘NESB’ to indicate that patients from this group might be disadvantaged in terms of access to appropriate health care facilities specifically due to language and cultural barriers.

### Questionnaire

We developed a questionnaire in the English language with questions adapted from validated questionnaires [2,15,16]. The questionnaire was pilot tested prior to use (see Additional file 1). In addition to questions about age, residency background, education and income the patients were asked about their visit to the hospital. They were asked specifically whether they considered contacting the GP before attending the ED and if not why they chose to come to the ED. The patients were asked also where they would go if they developed the same condition in their birth country.

The procedure for answering the questionnaire was self-completion or a face-to-face interview by the principal author. If required interpretation of the questions was provided by either a family member or a health service provided interpreter.

The study received ethical approval from the Human Ethics Committee of both the Princess Alexandra Hospital (HREC/12/QPAH/185) and the Queensland University of Technology (1200000369).

### Sample size and data analysis

Utilizing a two-tailed alpha of 0.05 and a beta of 0.10, we estimated that we would require 150 NESB patients and 590 ESB patients for the study to have 90% power to identify a significant difference between the groups [17].

Descriptive statistics were used to describe the demographic characteristics of the three groups. Pearson chi-square test was used to compare groups and multivariate logistic regression analyses was undertaken to examine the potential confounding effects of socioeconomic factors.

Analyses were performed using Statistical Package for the Social Sciences (SPSS) version 19 (IBM SPSS Statistics 19).

## Results

A total of 828 patients—446 (53.9%) ESB-BA, 151 (18.2%) ESB-NBA and 231 (27.9%) NESB—were interviewed during the study period. Table 1 provides the demographic characteristics of the study population, showing differences in educational levels and fortnightly income between the two groups.

**Table 1:** **Demographic characteristics of the participants, n (%)**

**Variable**	**ESB-BA**	**ESB-NBA**	**NESB**	**Total**
	**(n = 446)**	**(n = 151)**	**(n = 231)**	**(n = 828)**
**Sex**				
Females	204 (45.7)	57 (37.7)	102 (44.2)	363 (43.8)
Males	242 (54.3)	94 (62.2)	129 (55.8)	465 (56.2)
**Age (years)**				
18–39	217 (48.7)	50 (33.1)	118 (51.1)	385 (46.5)
40–64	173 (38.8)	67 (44.4)	76 (32.9)	316 (38.2)
65+	56 (12.6)	34 (22.5)	37 (16.0)	127 (15.3)
**Education (highest)**				
Did not complete secondary school	98 (22.0)	21 (13.9)	49 (21.2)	167(20.3)
Completed secondary school	170 (38.1)	70 (46.4)	54 (23.4)	287 (35.5)
Tertiary education	178 (39.9)	59 (39.7)	128 (55.4)	365 (44.2)
**Fortnight income (AUD$)**				
400–999	142 (31.8)	35 (23.2)	103(44.6)	280 (33.8)
1,000–1,499	101 (22.6)	35 (23.2)	52 (22.5)	188 (22.7)
1,500–1,999	80 (17.9)	27 (17.9)	34 (14.7)	141 (17.0)
2,000+	123 (27.6)	54 (35.8)	42 (18.2)	209 (26.4)

(Sex p = .230, Age p = .001, Education p < .001, Income p < .001).

Table 2 reveals that there was no significant differences between the types of visa and considering to contact a GP among NESB patients (p = .189). However, NESB patients who had been in Australia less than 2 years were least likely to consider contacting a GP 7(15.7%, 95% CI 7.8–28.8) compared to those who had been in Australia for more than 5 years 67 (47.5%, 95% CI 39.5–55.8), p = .001 (Table 3).

**Table 2:** **NESB patients considered contacting a GP and their visa status, n (%, 95% CI)**

**Variable**	**Yes**	**No**	**Total (%)** **(n = 231)**
**Visa status**			
Skilled migrant	18 (46.2, 31.6–61.4)	21 (53.8, 38.6–66.4)	39 (16.9, 12.6–22.3)
Refugee (Humanitarian)	22 (36.1, 25.2–48.6)	39 (63.9, 51.3–74.8)	61(26.4, 21.1–32.4)
Student	12 (31.6, 19.1–47.5)	26 (68.4, 52.5–80.9)	38 (16.5, 12.2–21.8)
Family, spouse	34 (48.6, 37.3–60.1)	36 (51.4, 40.0–62.8)	70 (30.3, 24.7–36.5)
Other (working, holiday, not sure)	6 (26.1, 12.6–46.5)	17 (73.9, 53.5–87.4)	23 (10.0, 6.7–14.5)

p = .189.

**Table 3:** **Patients considered contacting a GP and length of stay in Australia, n (%, 95% CI)**

**Length of stay in Australia**	**NESB ** **(n = 231)**			**ESB-NBA ** **(n = 150)**		
	**Yes**	**No**	**Total**	**Yes**	**No**	**Total**
	7	38	45	6	4	10
Less than 2 years	(15.7, 7.8–28.8)	(84.4,71.2–92.3)	(19.5,14.9–25.1)	(60.0,31.3–83.2)	(40.0, 16.8–68.7)	(6.7, 3.7–11.8)
	18	27	45	23	12	35
2–4 years	(40.0,27.0–54.6)	(60.0,45.5–73.0)	(19.5,14.9–25.1)	(65.7,49.2.4–79.2)	(34.3, 20.8–50.9)	(23.3,17.3–30.7)
	67	74	141	68	37	105
5 years or more	(47.5,39.5–55.8)	(52.5, 44.3–60.1)	(61.0,54.6–67.1)	(64.8,55.3 –73.2)	(35.2, 26.8–44.8)	(70.0,62.2–76.8)

P = .001 for NESB / P = .945 for ESB-NAB.

Compared to the ESB-BA patients, the NESB patients were less likely to consider contacting a GP before attending the ED (OR 0.6, 95% CI 0.4–0.8), however ESB-NBA patients were more likely to consider contacting a GP (OR 1.7, 95% CI 1.1–2.5) (Table 4). Both the NESB patients and the ESB-NBA patients were significantly more likely than the ESB-BA patients to report not having a GP and less likely to think that the ED could deal with their problem better than a GP (Table 4). NESB patients also reported that the belief that it would take a long time to see a GP as the reason for coming to the ED (OR 6.2, 95% CI 3.7–10.4) (Table 4).

**Table 4:** **Logistic regression model for considered contacting a GP & main reasons for attending the ED**

**Reasons for attending the ED**	**OR (95% CI)**	***P***	**OR (95% CI)**	***P***
**Reference group: ESB-BA**				
	**NESB**		**ESB-NAB**	
Considered contacting a GP before visiting the ED	0.6 (0.4–0.8)	0.002	1.7 (1.1–2.5)	0.013
I do not have a GP	7.9 (4.7–13.4)	<0.001	2.2 (1.1–4.4)	0.038
GPs charge extra fees	1.0 (0.4–2.1)	0.934	0.6 (0.2–1.9)	0.356
My GP’s opening hours are not suitable	1.9 (0.9–4.0)	0.117	0.6 (0.2–2.0)	0.357
It would take a long time to get an appointment with my GP	6.2 (3.7–10.4)	< 0.001	0.4 (0.1–1.4)	0.152
The ED is closer than my GP	0.4 (0.9–1.5)	0.182	0.2 (0.0–1.4)	0.107
The ED can deal with the problem better than a GP	0.5 (0.3–0.8)	0.001	0.7 (0.3–0.9)	0.022
I generally prefer the ED than a GP	1.6 (0.6–4.0)	0.306	1.0 (0.3–3.3)	0.973

Table 5 shows where overseas born patients would seek help if they developed the same problem in their birth countries. More than 30% of ESB-NBA and 50% of NESB said that they would visit a primary care service.

**Table 5:** **Where would immigrants seek help if they developed the same problem in their birth country, n (%)**

	**Hospital ED**	**GP**	**Private doctor**	**Other**	**Total**
ESB-NBA	98 (68.5)	41 (28.7)	4 (2.8)	0 (0)	143 (100)
NESB	109 (47.4)	45 (19.6)	71 (30.9)	5 (2.2)	230 (100)
Total	216 (55.5)	86 (23.1)	75 (20.1)	5 (1.3)	373 (100)

## Discussion

The study showed that the NESB patients in triage categories 3 to 5 were far less likely than those patients with an ESB to consider contacting a GP before attending the ED. Their reported reasons for not contacting a GP were either that they do not have a regular GP or that it can take a long time to obtain an appointment with their GP. However, the ESB-BA patients were more likely than the NESB patients and the ESB-NBA patients to perceive the ED as more suitable for treating their medical condition.

In our sample, over 50% of the NESB and ESB-NBA patients indicated that they would go to a GP or private doctor if they were to develop the same condition in their country of origin. Thus, the study findings suggest that the NESB patients did not choose the ED according to the urgency of their medical condition or the belief that the ED would deal better with their problem than a GP. However, there is a possibility that some of these patients attended the ED because they thought their condition would not need a GP so did not secure one. It is also possible that the condition was considered to be a temporary or transient one which could be addressed in the ED without the need to register with a GP.

These results agree with other studies which have indicated that immigrants and linguistic and ethnic minorities tend to use ED services for non-urgent conditions at the expense of primary health care [1,2,18]. A Danish study reported immigrants were more likely to have irrelevant ED visits than the Danish population [2]. Furthermore, an American study found that a large number of Somali patients use the ED for care that a GP normally provides [1]. Similarly, Cots and colleagues suggested that immigrants in Spain tend to use the ED in preference to other health services [19].

Our findings suggested that patients who have been in Australia for a short period of time are less likely to consider contacting a GP before attending the ED might support the existence of some barriers such as the unawareness of service availability and a lack of knowledge about the health system in the new country. The short stay of these groups in the country would also make it difficult for them to establish a relationship with any primary health care services. The majority of NESB patients in the study also thought it takes too long to obtain an appointment with a GP; this might be due to unfamiliarity to this way to access health care in their countries of origin. We suggest that educational intervention regarding the importance of having a regular GP, how to make an appointment with a GP, availability of health services and the health system in Australia, targeting new arrivals to the country, which might help them to access the most appropriate services to meet their health needs. Such education might reduce the burden on already overcrowded EDs in Australia [20].

Despite the insight that it provided on the differences between NESB and ESB patients’ use of ED services, this study had several limitations. First, the use of a convenience sample and the cross-sectional nature of the study limit the generalisability of the findings as there might be some over representations for certain overseas born group. The generalisability of the study is also limited based on the healthcare system. Second, the study period was limited to the four-month data collection period. Third, we assumed that country of birth define the background which might not be the case all times. Finally, we considered NESB patients as one group. However, people from NESBs are not a homogeneous group; their attitudes towards ED services might differ according to their ethnicity and cultural needs.

## Conclusions

The study showed that NESB patients in our study area might attend the ED for other reasons besides an urgent medical condition. Patients who are new to Australia and those on student or refugee visas are less likely to consider contacting a GP before attending the ED. These groups might be unfamiliar with their options within the Australian health care system. Therefore, educational interventions might assist them accessing the most appropriate health care service that meet their needs and thus reduce the burden on the hospital ED.

### Consent

Written informed consent was obtained from the patient for the publication of this report and any accompanying images.

## Additional file

Additional file 1:
**Emergency Department Questionnaire.**

## Abbreviations

ABS: Australian Bureau of Statistics
ATS: Australasian Triage Scale
CALD: Culturally and Linguistically Diverse
ED: Emergency Department
ESB-BA: English Speaking Background Born in Australia
ESB-NBA: English Speaking Background Not Born in Australia
GP: General Practitioner
PAH: Princess Alexandra Hospital
NESB: Non-English Speaking Background
