# Supplementary material for: Subjective reasons why immigrant patients attend the emergency department
Source: BMC Emerg Med. 2015 Mar 28;15:4. doi: 10.1186/s12873-015-0031-8 (PMC4378552; doi:10.1186/s12873-015-0031-8)
Supplement: Additional file 1: — Emergency Department Questionnaire. [file 12873_2015_31_MOESM1_ESM.docx]

**Emergency Department Questionnaire**

**Section 1:** **Socio-demographic factors**

1. Are you male or female?  Male  Female
2. Date of birth _______________________________

or age _________________________

1. Country of birth _______________________________
2. Language spoken at home _______________________________
3. Educational level  Did not complete secondary school

Completed secondary school only

Higher Education

1. Household income per fortnight

$400–999  $1000–1499  $1500–1999  $2000+

**If you were born in Australia or from English speaking background go to section 2 (next page)**

1. How long have you lived in Australia

Less than 2 years  2-4 years  5 years or more

1. What visa category did you enter Australia on?  Skilled migrant visa

Refugee (Humanitarian) visa

Student visa

Family, spouse visa

Other, please specify ____________

1. How well do you speak English?

Excellent  Very good  Good  Poor  Not at all

1. Are you going to use a professional interpreter at the Emergency Department (ED)?

Yes  No

1. Are you going to use a family member or a friend as an interpreter at the ED?

Yes  No

1. Were you offered the services of an interpreter, upon your arrival to the ED?

Yes  No

1. Do you know that you could have the services of a professional interpreter, free of charge, for your visit to the ED?

Yes  No

**Section 2: Reasons of ED service use**

**14.** What is the main problem brought you to the ED today? _______________________________________________________________________

**15.** How would you define your problem today?

Emergency (need to be seen immediately)

Urgent (can wait for 10-30 minutes)

Semi-urgent (can wait for 2 hours)

Non-urgent (can wait for more than 2 hours)

**16.** If you were living in your birth country and developed the same illness where would you be most likely to seek medical care? (Overseas born only)

Hospital Emergency

General practitioner (GP)

Private Doctor

Other, please specify ___________________________________________

**17.** Did you consider contacting your GP instead of coming to ED today?

Yes (Go to next question)

No, why did you choose ED? (Choose one or more from below)

I do not have a GP

GPs charge extra fees

My GP does not speak my language

It would take long time to get an appointment with GP

My GP opening hours are not suitable

The ED is closer than my GP

The ED can deal with the problem better than my GP

I generally prefer the ED than GP

Other, please specify _____________________________________________

**18.** Do you know how to make a call for emergency medical help?

Yes  No

**19.** Are you afraid to call for an ambulance, when required?

Yes  No

**20.** Do you think the Ambulances charge extra fees for service?

Yes  No  Not sure

**21.** Have you ever called for an ambulance?

Yes  No

**22.** Do you find it difficult to explain your problem on the telephone?

Yes  No

**Section 3: Satisfaction among patients for today’s visit to ED**

**(**Fill this part after ED care completed and before leaving ED)

| How would you rate: | **Excellent** | **Very good** | **Good** | **Fair** | **Poor** |
| --- | --- | --- | --- | --- | --- |
| **23.** The skills with which the staff assisted you. |  |  |  |  |  |
| **24.** The nursing staff’s interest in you and your medical condition. |  |  |  |  |  |
| **25.** The amount of time you had with the staff during your stay. |  |  |  |  |  |
| **26.** The attention given to your emotional and spiritual needs. |  |  |  |  |  |
| **27.** The staff’s encouragement for you to talk freely about your condition, concerns, or fears. |  |  |  |  |  |
| **28.** The nursing staff’s responsiveness in managing your pain needs. |  |  |  |  |  |
| **29.** Staff concern for your well-being. |  |  |  |  |  |
| **30.** Staff respect for your privacy. |  |  |  |  |  |
| **31.** Staff courtesy and friendliness. |  |  |  |  |  |
| **32.** The promptness of service. |  |  |  |  |  |
| **33.** The communication with staff or professional interpreter (if used)? |  |  |  |  |  |
| **34.** The amount of time you had with the doctor. |  |  |  |  |  |
| **35.** The doctor’s explanation regarding your medical tests and procedures. |  |  |  |  |  |
| **36.** The overall quality of the care you received. |  |  |  |  |  |
| **37.** How well the care met your expectations. |  |  |  |  |  |
| **38.** Your satisfaction with the care and service provided to you. |  |  |  |  |  |

**39.** Would you return to the same ED if you have another problem that required emergency care?  Yes  No

**40.** What do you think is the most important element for your ED care?

________________________________________________________________________

**Comments:** ________________________________________________________________________
